# A collection of bioconductor methods to visualize gene-list annotations

**DOI:** 10.1186/1756-0500-3-10

**Published:** 2010-01-19

**Authors:** Gang Feng, Pan Du, Nancy L Krett, Michael Tessel, Steven Rosen, Warren A Kibbe, Simon M Lin

**Affiliations:** 1 Northwestern University Biomedical Informatics Center (NUBIC, part of the Northwestern CTSA) and The Robert H. Lurie Comprehensive Cancer Center, Northwestern University, Chicago, IL 60611, USA

## Abstract

**Background:**

Gene-list annotations are critical for researchers to explore the complex relationships between genes and functionalities. Currently, the annotations of a gene list are usually summarized by a table or a barplot. As such, potentially biologically important complexities such as one gene belonging to multiple annotation categories are difficult to extract. We have devised explicit and efficient visualization methods that provide intuitive methods for interrogating the intrinsic connections between biological categories and genes.

**Findings:**

We have constructed a data model and now present two novel methods in a Bioconductor package, "GeneAnswers", to simultaneously visualize genes, concepts (a.k.a. annotation categories), and concept-gene connections (a.k.a. annotations): the "Concept-and-Gene Network" and the "Concept-and-Gene Cross Tabulation". These methods have been tested and validated with microarray-derived gene lists.

**Conclusions:**

These new visualization methods can effectively present annotations using Gene Ontology, Disease Ontology, or any other user-defined gene annotations that have been pre-associated with an organism's genome by human curation, automated pipelines, or a combination of the two. The gene-annotation data model and associated methods are available in the Bioconductor package called "GeneAnswers " described in this publication.

## Findings

The gene list from a microarray study is usually summarized by Gene Ontology [1] or Disease Ontology [2] annotations to provide a higher-level understanding of the functionalities of the genes identified in such an experiment. We explored the existing methods available in Bioconductor http://www.bioconductor.org to visualize the annotation results, and then extended those methods to create the GeneAnswers package.

### The Gene-annotation Data Model

Formally, the annotation  is a mapping between the two sets: genes G and concepts C,

Note that these mappings are usually many-to-many, i.e., one gene belongs to multiple concepts and one concept includes multiple genes.

Accordingly, we implement the following GeneAnswers class in our Bioconductor package of "GeneAnswers " (Table 1).

**Table 1:** The GeneAnswers Class

GeneAnswers Class	
Slots:	
* Category list ()	
* Gene list ():	Gene IDs (required)
	Fold Change (optional)
	Expression profile (optional)
	Hypergeometric test result (calculated) ()
Methods:	
* Barplot	
* Concept-and-gene network	
* Concept-and-gene cross tabulations	

The GeneAnswers package calls different modules to generate the figures depending on the users' requirements.

### Testing Data Set

The testing dataset was obtained from a microarray experiment running on Affymetrix human HG-U133A+ version 2.0 chips for a multiple myeloma cell line treated with dexamethasone for 24 hours (three biological replicates). Dexamethasone is a synthetic glucocorticoid. Glucocorticoids are used to treat several hematologic malignancies including multiple myeloma, however the mechanism of action is not completely understood. The gene list from the microarray experiment can help us gain a better understanding of the glucocorticoid-induced gene regulation in myeloma cell lines.

In this dataset, there were 319 genes identified as significantly differentially expressed, based on cut-off criteria of fold change more than 2 and False Discovery Rate (FDR) less than 0.01. Using our new Bioconductor package - GeneAnswers, a hypergeometic test [3] for over-represented Gene Ontology (GO) terms of the input gene list was conducted. The final GeneAnswers output, describing the GO analysis results of the input gene list (including GO identifiers, GO terms, gene numbers, hypergeometric test p-values and genes), was generated and the results sorted by hypergeometic test p-values. Six relevant GO terms were selected for further analysis based on their biological importance and statistical significance. This testing data set is included in the GeneAnswers package as an example.

### Barplots

**Figure 1:**
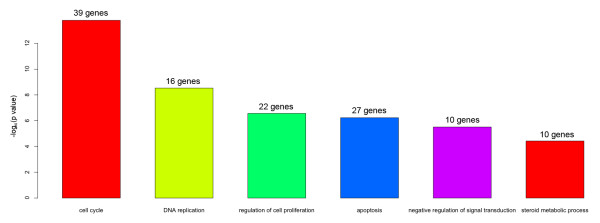
**Distribution of statistical significance on selected GO categories**. P-values were calculated from the hypergeometric test. The number of genes in each category is also listed.

As discussed above, we selected six GO terms and tested their significance in the list of 319 genes via a hypergeometic test. The result is shown as a barplot in Figure 1, which includes the test statistics and the number of genes in each category. However, the barplot cannot provide more details about additional relationships between the genes and the GO terms. For instance, the gene DHCR24 (full name: 24-dehydrocholesterol reductase) is involved in three biologically relevant GO categories (cell cycle, apoptosis and steroid metabolism). From the barplot, it is not clear how many genes belong to multiple categories.

### Concept-and-Gene Networks

One of the most important contributions of the GeneAnswers package is to formally introduce the GeneAnswers class (the concept-to-gene mapping class) into Bioconductor. With this class, the package can visually represent the relationship between genes and any given concepts (gene ontology, disease ontology, and etc.) in two different ways: a concept-and-gene-network, highlighting the involvement of a gene in multiple annotation concepts; and the concept-and-gene cross tabulation, which enables a more traditional heat map visualization with annotations.

**Figure 2:**
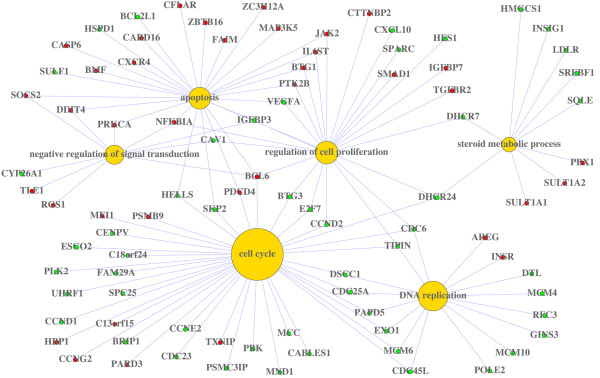
**Concept-and-gene network**. Green and red solid circles represent down and up regulation of genes, respectively. Yellow solid circles stand for the given GO categories. The size of each yellow dot is proportional to the statistical significance of the identified genes in corresponding GO categories.

The concept-and-gene network shows the complex relationship between the genes and the given concepts (Figure 2). It can also include more information, such as gene expression levels, by adding colors to the gene symbols. This type of presentation can visually highlight the key genes that are involved in several different concepts. Unlike the previous barplot, one can easily find the gene DHCR24 involved in three different categories: cell cycle, apoptosis and steroid metabolic process in Figure 2.

Researchers might be more interested in the relationship between gene expression profile and relative GO terms. Therefore, the GeneAnswers package also supplies another way, the "concept-and-gene cross tabulation", to show such details.

### Concept-and-Gene Cross Tabulations

**Figure 3:**
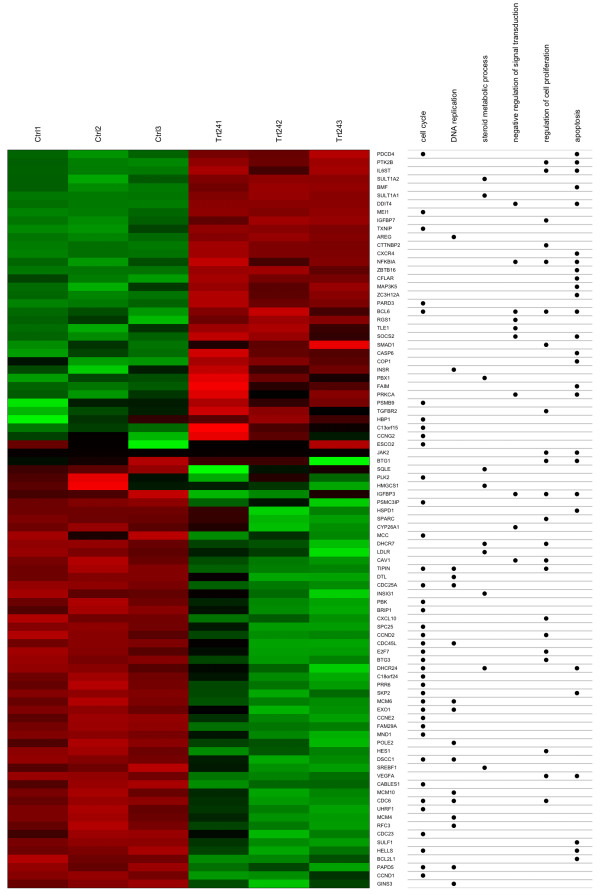
**Concept-and-gene cross tabulations**. The left side is a heatmap in which green and red bars represent lower and higher expression based on the gene expression profile, respectively. The right side is the annotation table, which shows the relationship between genes and six relevant GO categories. Ctrl: control; Trt24: treatment after 24 hours.

The "concept-and-gene cross tabulation" combines the traditional heat map with the annotation table. For example, researchers can identify that DHCR24, down regulated in the treatment samples, is related to three relevant biological processes (Figure 3). Note that the order of the concepts (columns on the right side) can be rearranged by clustering the presence/absence matrix of the genes associated with each concept; users have the option to turn on or off the clustering.

Users can combine these two types of visualization tools to identify which genes and biological pathways are potentially involved in the treatment effects. For instance, it is clear from Figure 2 and Figure 3 that genes associated with DNA replication- such as CDC6, CDC45L, CDC25A, MCM4, MCM10, and MCM6 - are all down regulated after dexamethasone treatment.

## Conclusion

The "Concept-and-Gene Network" and the "Concept-and-Gene Cross Tabulation" visualization methods provided by the new Bioconductor package "GeneAnswers" are powerful tools that generate a macroscopic view for investigators to understand the relationships between a given gene list and relevant annotations. In addition to seeing an easy to understand blueprint for all genes and statistically significant annotation categories, researchers are also able to identify key genes involved in several different categories and visualize how the gene expression patterns relate to each potential pathway and biological functions. These visualization methods can be incorporated into large-scale genomic data processing pipelines. As of this release, the GeneAnswers Bioconductor package only generates static images. As the number of genes reaches to the magnitude of thousands, the gene symbols and concept categories in the figure will be cramped, although the overall structure can be legible. Such a limitation can be addressed by interactive figures with zooming or scrolling capabilities.

## Availability and requirements

Project name: GeneAnswers

Project home page: http://bioconductor.org/packages/2.5/bioc/html/GeneAnswers.html

Operating system(s): Platform independent

Programming language: R version 2.9.0 or higher

Other requirements: annotate 1.20.0, AnnotationDbi 1.6.0, Biobase 2.2.0, DBI 0.2.4, GO.db 2.2.11, KEGG.db 2.2.11, the most updated Megadata packages, org.Hs.eg.db, org.Mm.eg.db, org.Rt.eg.db in Bioconductor and igraph 0.5.1, RSQLite 0.7.1, XML 1.98.1, xtable 1.5.4 Heatplus 1.12.0, MASS 7.2.44, RColorBrewer 1.0.2 in R

## Competing interests

The authors declare that they have no competing interests.

## Authors' contributions

GF, DP, SL and WK participated in the GeneAnswers package coding and drafted the manuscript. NK, MT, and SR designed and conducted the Affymetrix experiment. All authors read and approved the final manuscript.
